# Differential Immunodominance Hierarchy of CD8^+^ T-Cell Responses in HLA-B*27:05- and -B*27:02-Mediated Control of HIV-1 Infection

**DOI:** 10.1128/JVI.01685-17

**Published:** 2018-01-30

**Authors:** Emily Adland, Matilda Hill, Nora Lavandier, Anna Csala, Anne Edwards, Fabian Chen, Marek Radkowski, Justyna D. Kowalska, Dimitrios Paraskevis, Angelos Hatzakis, Humberto Valenzuela-Ponce, Katja Pfafferott, Ian Williams, Pierre Pellegrino, Persephone Borrow, Masahiko Mori, Jürgen Rockstroh, Julia G. Prado, Beatriz Mothe, Judith Dalmau, Javier Martinez-Picado, Gareth Tudor-Williams, John Frater, Anette Stryhn, Soren Buus, Gustavo Reyes Teran, Simon Mallal, Mina John, Susan Buchbinder, Gregory Kirk, Jeffrey Martin, Nelson Michael, Jacques Fellay, Steve Deeks, Bruce Walker, Santiago Avila-Rios, David Cole, Christian Brander, Mary Carrington, Philip Goulder

**Affiliations:** a Department of Paediatrics, University of Oxford, United Kingdom; b Department of GU Medicine, The Churchill Hospital, Oxford University NHS Foundation Trust, Oxford, United Kingdom; c Department of Sexual Health, Royal Berkshire Hospital, Reading, United Kingdom; d Department of Immunopathology of Infectious and Parasitic Diseases, Hospital for Infectious Diseases, Medical University of Warsaw, Warsaw, Poland; e Medical School, National and Kapodistrian University of Athens, Athens, Greece; f Centre for Research in Infectious Diseases, National Institute of Respiratory Diseases, Mexico City, Mexico; g Nuffield Department of Medicine, University of Oxford, Oxford, United Kingdom; h Centre for Sexual Health and HIV Research, Mortimer Market Centre, London, United Kingdom; i Department of Medicine I, University Hospital Bonn, Bonn, Germany; j AIDS Research Institute IrsiCaixa, Institut d'Investigació en Ciències de la Salut Germans Trias i Pujol (IGTP), Universitat Autònoma de Barcelona, Badalona, Spain; k University of Vic-Central University of Catalonia (UVic-UCC), Vic, Barcelona, Spain; l Institució Catalana de Recerca i Estudis Avançats (ICREA), Barcelona, Spain; m Department of Paediatrics, Imperial College, London, United Kingdom; n Oxford Martin School, University of Oxford, Oxford, United Kingdom; o Department of Immunology and Microbiology, University of Copenhagen, Copenhagen, Denmark; p Department of Medicine, Vanderbilt University Medical Center, Nashville, Tennessee, USA; q Institute of Immunology and Infectious Diseases, Murdoch University, Perth, Australia; r San Francisco Department of Public Health, HIV Research Section, San Francisco, California, USA; s Department of Epidemiology, Johns Hopkins University, Bloomberg School of Public Health, Baltimore, Maryland, USA; t Department of Epidemiology and Biostatistics, University of California, San Francisco, California, USA; u U.S. Military HIV Research Program, Walter Reed Army Institute of Research, Silver Spring, Maryland, USA; v School of Life Sciences, EPFL, Lausanne, Switzerland; w Ragon Institute of MGH, MIT and Harvard, Boston, Massachusetts, USA; x Cardiff University School of Medicine, Heath Park, Cardiff, United Kingdom; y Immunocore Limited, Abingdon, Oxfordshire, United Kingdom; z Cancer and Inflammation Program, Leidos Biomedical Research, Frederick National Laboratory for Cancer Research, Maryland, USA; Ulm University Medical Center

**Keywords:** CD8^+^ T cell, HIV Gag, HIV Nef, HLA, HLA-B*27, human immunodeficiency virus

## Abstract

The well-characterized association between HLA-B*27:05 and protection against HIV disease progression has been linked to immunodominant HLA-B*27:05-restricted CD8^+^ T-cell responses toward the conserved Gag KK10 (residues 263 to 272) and polymerase (Pol) KY9 (residues 901 to 909) epitopes. We studied the impact of the 3 amino acid differences between HLA-B*27:05 and the closely related HLA-B*27:02 on the HIV-specific CD8^+^ T-cell response hierarchy and on immune control of HIV. Genetic epidemiological data indicate that both HLA-B*27:02 and HLA-B*27:05 are associated with slower disease progression and lower viral loads. The effect of HLA-B*27:02 appeared to be consistently stronger than that of HLA-B*27:05. In contrast to HLA-B*27:05, the immunodominant HIV-specific HLA-B*27:02-restricted CD8^+^ T-cell response is to a Nef epitope (residues 142 to 150 [VW9]), with Pol KY9 subdominant and Gag KK10 further subdominant. This selection was driven by structural differences in the F pocket, mediated by a polymorphism between these two HLA alleles at position 81. Analysis of autologous virus sequences showed that in HLA-B*27:02-positive subjects, all three of these CD8^+^ T-cell responses impose selection pressure on the virus, whereas in HLA-B*27:05-positive subjects, there is no Nef VW9-mediated selection pressure. These studies demonstrate that HLA-B*27:02 mediates protection against HIV disease progression that is at least as strong as or stronger than that mediated by HLA-B*27:05. In combination with the protective Gag KK10 and Pol KY9 CD8^+^ T-cell responses that dominate HIV-specific CD8^+^ T-cell activity in HLA-B*27:05-positive subjects, a Nef VW9-specific response is additionally present and immunodominant in HLA-B*27:02-positive subjects, mediated through a polymorphism at residue 81 in the F pocket, that contributes to selection pressure against HIV.

**IMPORTANCE** CD8^+^ T cells play a central role in successful control of HIV infection and have the potential also to mediate the eradication of viral reservoirs of infection. The principal means by which protective HLA class I molecules, such as HLA-B*27:05 and HLA-B*57:01, slow HIV disease progression is believed to be via the particular HIV-specific CD8^+^ T cell responses restricted by those alleles. We focus here on HLA-B*27:05, one of the best-characterized protective HLA molecules, and the closely related HLA-B*27:02, which differs by only 3 amino acids and which has not been well studied in relation to control of HIV infection. We show that HLA-B*27:02 is also protective against HIV disease progression, but the CD8^+^ T-cell immunodominance hierarchy of HLA-B*27:02 differs strikingly from that of HLA-B*27:05. These findings indicate that the immunodominant HLA-B*27:02-restricted Nef response adds to protection mediated by the Gag and Pol specificities that dominate anti-HIV CD8^+^ T-cell activity in HLA-B*27:05-positive subjects.

## INTRODUCTION

HLA-B*27:05 is strongly associated with slow progression in human immunodeficiency virus (HIV) infection (1–3). It has been proposed that it is the particular HIV type 1 (HIV-1)-specific CD8^+^ T-cell responses restricted by HLA-B*27:05 that provide a likely mechanism for protection. HLA-B*27:05 mediates an immunodominant response toward an epitope in p24 Gag, KK10 (KRWIILGLNK [residues 263 to 272]). HLA-B*27 has a unique structure among HLA-B class I molecules in having an absolute requirement for arginine at position 2 (P2) in the binding peptide (4). Loss of immune control and progression to AIDS in HIV-infected HLA-B*27:05-positive individuals appeared to be precipitated by selection of an escape mutation at Gag residue 264, most commonly R264K or R264G (5–7). This R264X escape mutant is selected prior to, and not as a result of, the sharp rise in viral load (6). The Arg-264 replacement results in reduced binding of the epitope to HLA-B*27:05 and reduced recognition of virus-infected cells.

More recently, an HLA-B*27:05-restricted Pol epitope, KY9 (KRKGGIGGY [residues 901 to 909]), has been described (8). The magnitude of responses to this epitope is only marginally lower than that to KK10 (8). At position 908 (P8) within the KY9 epitope, a viral escape mutant with an amino acid change from glycine to glutamic acid emerges soon after the R264X mutation in the Gag KK10 epitope. This would imply that KK10 and KY9 impose selection pressure on the virus at the same time and highlights KY9 as a contributing factor to HLA-B*27:05-mediated immune control of HIV.

The most prevalent subtype of HLA-B*27 worldwide is HLA-B*27:05, although many other natural variants of the molecule have been described to date, from HLA-B*27:01 to -B*27:99 (http://www.ebi.ac.uk/ipd/imgt/hla/align.html). Most of these differences have a direct impact on the peptide binding groove and therefore on the nature of the peptides binding that particular HLA-B*27 subtype. The particular peptides presented by these subtypes could potentially explain differences in disease susceptibility. HLA-B*27 has been especially well studied because of its strong association with ankylosing spondylitis (AS), and it is noteworthy that some HLA-B*27 subtypes are associated with AS and others are not. For example, HLA-B*27:01, -B*27:02, -B*27:04, -B*27:05, -B*27:07, and -B*27:08 have been linked with AS, whereas HLA-B*27:06 and -B*27:09 are not associated with AS (9).

HLA-B*27:02 reportedly is present in 1 to 10% of HLA-B*27-positive subjects in Northern Europe, 20% in Spain and Portugal, 35 to 50% in Greece, and ∼55% in Arab and Jewish populations (9–11). However, in Germany this figure is 14% in a population where HLA-B*27 prevalence is ∼10% (12), and in Poland this figure is 26 to 29% in a population where HLA-B*27 prevalence is ∼14% (13).

As stated above, there are three amino acid differences between HLA-B*27:05 and HLA-B*27:02. These 3 amino acids line the F pocket of the peptide binding groove, therefore influencing the type of residue at the carboxy-terminal anchor position (PC). HLA-B*27:02 has asparagine at HLA position 77, whereas HLA-B*27:05 uses the negatively charged aspartate. The F pocket of HLA-B*27:02 is therefore lined with residues conferring a lower overall negative charge than HLA-B*27:05, and for this reason the peptide binding motif does not include basic residues (Lys or Arg) at PC in HLA-B*27:02-binding peptides, but these are a feature of many of the peptides binding to HLA-B*27:05. Additionally, HLA-B*27:05 has isoleucine at position 81, in contrast to alanine in HLA-B*27:02. Thus, the F pocket in HLA-B*27:02 is potentially slightly larger and may be able to accommodate amino acids with bulkier side chains. Together, these differences provided a unique opportunity to investigate the differences in the HIV epitopes presented by HLA-B*27:05 and HLA-B*27:02 and their respective roles in immune control of HIV.

## RESULTS

### HLA-B*27:02 is associated with protection against HIV disease progression.

**FIG 1:**
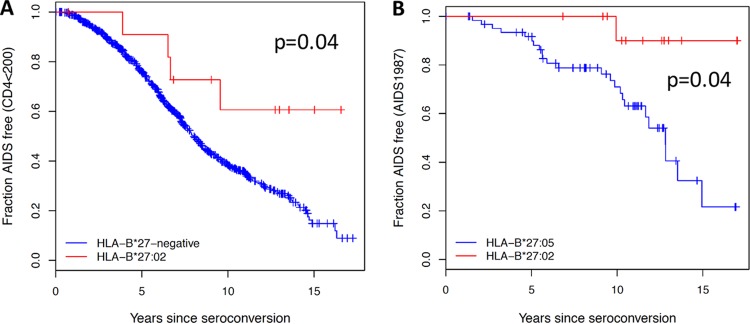
HLA-B*27:02 is associated with protection against HIV disease progression. HLA differences by log rank test are shown as follows: fraction of HLA-B*27:02-positive (*n* = 12) and HLA-B*27-negative (*n* = 721) subjects remaining AIDS free (absolute CD4 < 200/μl) (A) and fraction of HLA-B*27:02-positive (*n* = 13) and HLA-B*27:05-positive (*n* = 64) subjects remaining AIDS free by the 1987 CDC definition for AIDS.

In cohorts that have been studied to determine the impact of host genetic factors on HIV disease outcome, the prevalence of HLA-B*27:02 is relatively low, approximately 10-fold lower than that of HLA-B*27:05. Survival analyses were performed on seroconverter cohorts, and for all four outcomes studied, HLA-B*27:02 associated more strongly with slower progression than did HLA-B*27:05, though the sample sizes are very limited in the HLA-B*27:02 group (Table 1; Fig. 1). Both HLA-B*27:02 and HLA-B*27:05 were associated significantly with mean viral loads lower than those without these alleles, and there was no significant difference between the two alleles on viral control. Although, unexpectedly, in the current analyses HLA-B*27:05 was not significantly associated with slow disease progression, HLA-B*27:05 has been consistently and in many studies associated with control of HIV viremia, as well as with slow disease progression (1–3, 5). Our subtype-specific analyses, of both viral setpoint and disease progression, suggest slightly better protection in both cases conferred by HLA-B*27:02 than HLA-B*27:05. However, in a previous study of viral setpoint only, involving 2,767 subjects, also of European descent, suggested that HLA-B*27:05 (odds of being an immune controller versus progressor, 3.34) was slightly more protective than HLA-B*27:02 (odds ratio, 2.53) (2). Thus, these data together would indicate that HLA-B*27:02 is associated with protection against HIV disease progression and provides a degree of protection similar to or possibly even a somewhat greater than that provided by HLA-B*27:05.

**TABLE 1:**
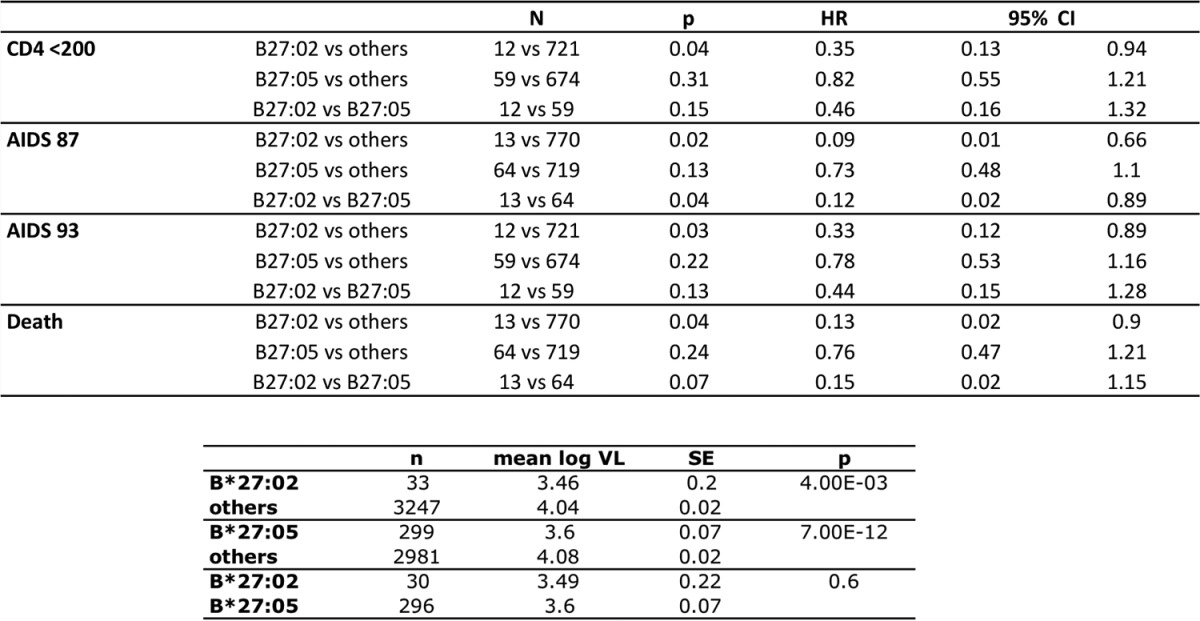
Protective effect of HLA-B*27:02^*a*^

a The top portion presents data from the Multicenter AIDS Cohort Study (MACS) showing comparisons between HLA-B*27-negative subjects (referred to as “others”) and HLA-B*27:02-positive subjects and HLA-B*27:05-positive subjects for time to CD4 count of <200 mm^3^, AIDS defined by the CDC 1987 criteria, AIDS defined by the CDC 1993 criteria, and death. Values for the Cox proportional hazards model are shown. HR, hazard ratio; CI, confidence interval. The bottom portion shows analysis of variance (ANOVA) for median viral loads (VLs) from the MACS cohort comparing HLA-B*27-negative, HLA-B*27:02-positive, and HLA-B*27:05-positive subjects.

### Nef VW9 is the immunodominant HLA-B*27:02-restricted CD8^+^ T-cell epitope.

**FIG 2:**
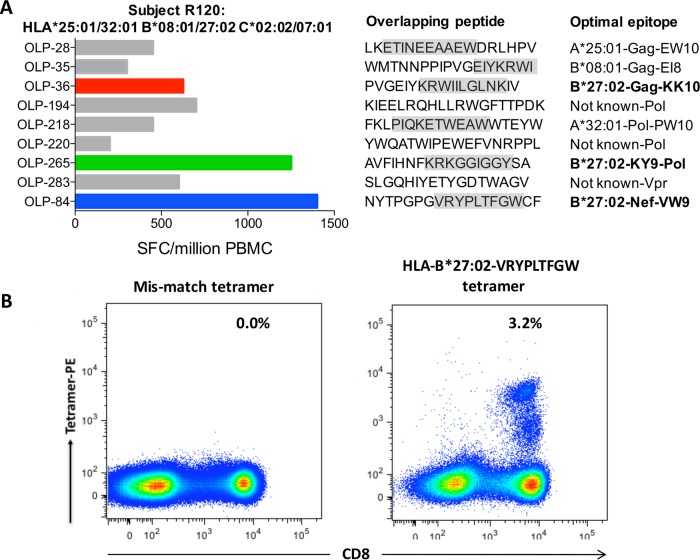
Characterization of HLA-B*27:02-restricted cytotoxic T-lymphocyte (CTL) responses. (A) IFN-γ ELISpot assay recognition of a panel of 410 overlapping peptides spanning the B clade proteome. The amino acid sequences of overlapping peptide and confirmed optimal epitope and HLA restriction are shown. Gag KK10 is KRWIILGLNLK, Pol KY9 is KRKGGIGGY, and Nef VW9 is VRYPLTFGW. (B) Confirmative fluorescence-activated cell sorter (FACS) staining of a HLA-B*27:02-positive subject R120 with an HLA-mismatched tetramer on the left and the HLA-B*27:02-restricted VRYPLTFGW (VW9) on the right.

**FIG 3:**
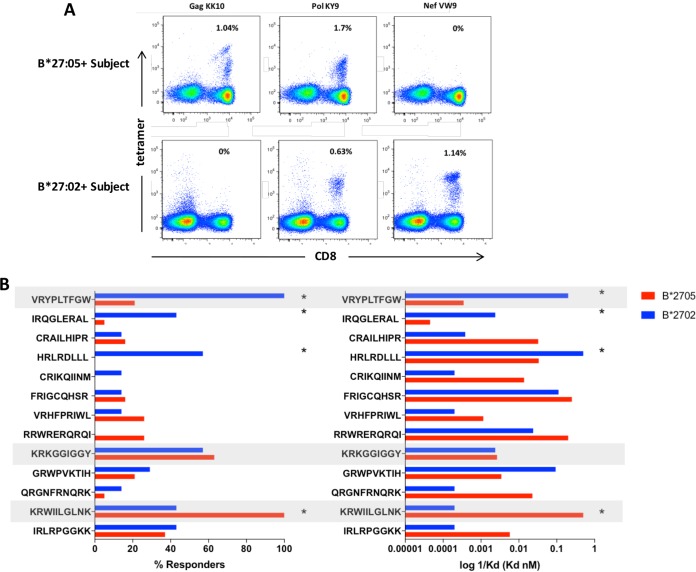
Differential recognition of HLA-B*27-restricted epitopes. (A) Epitope responses from one representative HLA-B*27:05-positive subject (top) and one HLA-B*27:02-positive subject (bottom). CD8 is shown on the *x* axis and tetramer-PE on the *y* axis. (B) (Left) Percentage of HLA-B*2702-positive subjects (*n* = 7) and HLA-B*2705-positive subjects (*n* = 19) making responses to the indicated epitopes. **, *P* < 0.001, Fisher's exact test. (Right) Differential recognition of HLA-B*27 epitopes is related to peptide-MHC binding avidity. Peptide-MHC binding avidity is measured as the Kd (equilibrium dissociation constant) and shown as log10 (1/Ka), where Ka is the equilibrium constant for dissociation of an acid. Shaded are the three epitopes Gag KK10 (KRWIILGLNLK), Pol KY9 (KRKGGIGGY), and Nef VW9 (VRYPLTFGW).

To characterize the HIV-specific CD8^+^ T-cell responses associated with HLA-B*27:02 and immune control of HIV infection, we tested in gamma interfereon (IFN-γ) enzyme-linked immunosorbent spot (ELISpot) assays recognition of a panel of 410 overlapping peptides spanning the B clade proteome (14). The immunodominant response among 7 HLA-B*27:02-positive subjects whose HIV-specific CD8^+^ T-cell responses were analyzed was to an epitope in Nef, defined using peptide-major histocompatibility complex (MHC) tetramers as the 9-mer VRYPLTFGW (Nef residues 133 to 141), confirmed via tetramer staining (Fig. 2). Summarizing the HLA-B*27-restricted CD8^+^ T-cell responses observed in these 7 HLA-B*27:02-positive subjects and in 19 HLA-B*27:05-positive subjects (Fig. 3A) confirms the reversal of the immunodominance pattern observed in HLA-B*27:05-positive subjects, in which Gag KK10 is dominant and the Pol KY9 response codominant or subdominant, and typically there is no HLA-B*27:05-restricted Nef-specific response. In the HLA-B*27:02-positive subjects, the Nef VW9 response was dominant, Pol KY9 subdominant, and Gag KK10 below Pol KY9 in the hierarchy (Fig. 3).

### HLA-B*27:05 and HLA-B*27:02 have distinct peptide binding preferences.

To help explain the differences observed in the CD8^+^ T-cell immunodominance patterns observed between HLA-B*27:02-positive and HLA-B*27:05-positive subjects, the peptide-MHC binding avidity was determined for each of the HLA-B*27-restricted epitopes. Consistent with the differences in peptide binding motifs between HLA-B*27:05 (inability to bind Trp and ability to bind basic residues Lys, Arg, and His in the F pocket) and HLA-B*27:02 (ability to bind Trp and inability to bind basic residues Lys, Arg, and His in the F pocket), we observed that the Gag KK10 epitope, which is immunodominant in HLA-B*27:05-positive subjects, bound poorly to HLA-B*27:02; conversely, the Nef VW9 epitope, which is immunodominant in HLA-B*27:02-positive subjects, did not bind well to HLA-B*27:05 (Fig. 3C). Overall it is striking that none of the B*27-restricted peptides tested that had a basic residue, Lys or Arg, at the carboxy-terminal position (PC) bound successfully to HLA-B*27:02: namely, Gag IK9, Gag KK10, Gag QK10, Pol FR9, and Env CR9. Also, as has been well described (15, 16), strong peptide-MHC binding avidity does not necessarily translate into high immunogenicity. For example, Env HI9 (HRLRDLLLI) binds well to both HLA-B*27:02 and HLA-B*27:05, but responses were detected only in subjects expressing HLA-B*27:02. Thus, adequate binding is a requirement for immunogenicity but is not sufficient to predict it.

### Structural modeling of the impact of HLA-B*27:05/HLA-B*27:02 polymorphisms on F-pocket amino acid compatibility.

**FIG 4:**
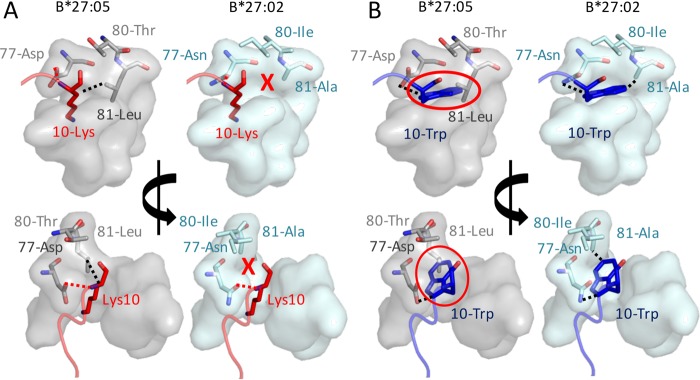
Structural modeling of the impact of HLA-*27:05/HLA-B*27:02 polymorphisms on F-pocket amino acid compatibility. The crystal structure of HLA-B*27:05 in complex with the KK10 peptide (17, 18) was used to model how the polymorphisms in HLA-B*2702 might impact C-terminal anchoring of peptide epitopes. Wincoot was used to generate a model with the following mutations in the HLA-B*27:05 F pocket: D77N, T81I, and L81A. The peptide was modeled with a Trp or a Lys at position 10. (A) The KK10 peptide is shown in red, with Lys-10 depicted by red sticks. Left images show the HLA-HLA-B*2705 F pocket in gray, with Asp-77, Thr-80, and Leu-81 depicted by gray sticks. The dotted line represents a van der Waals contact between Lys-10 and Leu-81. Right images show the modeled HLA-B*2702 F pocket in cyan, with Asn-77, Ile-80, and Ala-81 depicted by cyan sticks. A red cross represents the loss of interactions between Lys-10 and Ala-81 in HLA-B*27:02. (B) KK10 peptide modeled with Trp at position 10 is shown in blue, with Trp-10 depicted by blue sticks. Left panels show the HLA-B*2705 F pocket in gray, with Asp-77, Thr-80, and Leu-81 depicted by gray sticks. A red circle shows the steric clash that would occur between Trp-10 and Leu-81. Right images show the modeled HLA-B*2702 F pocket in cyan, with Asn-77, Ile-80, and Ala-81 depicted by cyan sticks. Black dotted lines represent a van der Waals contact, and red dotted lines represent hydrogen bonds.

The crystal structure of HLA-B*27:05 in complex with the KK10 peptide has been previously determined, but the crystal structure of HLA-B*27:02 has not been elucidated. Thus, the polymorphisms in the F pocket that distinguish HLA-B*27:05 from HLA-B*27:02 (17, 18) (D77N, T80I, and L81A) were modeled to explore the distinct C-terminal peptide (PC) residue preferences between the two HLA alleles (Fig. 4). KK10 epitope PC residue Lys-10 forms a “peg-in-hole”-type interaction in the HLA-B*27:05-KK10 structure, forming a stabilizing van der Waals interaction with HLA residue Leu-81. This interaction is likely disrupted in HLA-B*27:02 because of the smaller side chain at residue 81 (Ala in HLA-B*27:02 in contrast to Leu in HLA-B*27:05) (Fig. 4A). This polymorphism also results in the widening of the F pocket in HLA-B*27:02, which would tend to increase mobility of the Lys-10 side chain that could destabilize the peptide.

Additionally, HLA-B*27:05 possesses Asp at position 77, which provides a negative charge that is favorable for the binding of positively charged basic amino acids. In contrast, HLA-B*27:02 possesses Asn at position 77, which is neutral and less favorable for binding to basic amino acids. Although the structural modeling did not reveal an obvious difference in the abilities of Asp-77 and Asn-77 to form a hydrogen bond with the main chain N group of Lys-10 in the KK10 peptide, this difference in charge could also play a role in the differential selection of amino acids based on their C-terminal residues between HLA-B*27:05 and HLA-B*27:02. Together, these observations are consistent with the low avidity of KK10 peptide-MHC binding to HLA-B*27:02 (Fig. 3C) and our observation that KK10 is not the dominant HIV epitope in HLA-B*27:02-positive individuals (Fig. 3B).

We next modeled the interaction between HLA-B*27:05 and HLA-B*27:02 with Trp as the C-terminal peptide residue. The tighter F pocket in HLA-B*27:05 is unlikely to accommodate KK10 peptide PC 10-Trp because of a potential steric clash with HLA residue Leu-81. In contrast, the wider F pocket in HLA-B*27:02 is ideally suited to bind Trp, which could form van der Waals contacts with Ala-81 (Fig. 4B). These observations are consistent with the switch in immunodominant responses from KK10 in HLA-B*27:05 individuals to VW9 in HLA-B*27:02 individuals and demonstrate the extreme peptide selectivity of HLA alleles that differ at only a few key residues in the peptide binding groove.

### Immune escape selection pressure matches CD8^+^ T-cell immunodominance hierarchy.

A hallmark of disease progression in subjects expressing HLA-B*27:05 is the impact of escape within Gag KK10 as a precipitant. To date, progression to AIDS (CD4 < 200) has not been observed in an HLA-B*27:05-positive subject without escape occurring in this epitope. However, the kinetics of viral escape are strongly influenced by the immunodominance hierarchy of the CD8^+^ T-cell responses (19). Analysis of autologous viral sequences in HLA-typed subjects demonstrated that in HLA-B*27:05-positive subjects, the strongest selection pressure is imposed by the dominant Gag KK10 response, followed closely by the codominant or subdominant Pol KY9 response, and as expected, no selection pressure was evident within the Nef VW9 epitope (Table 2; odds ratios for escape in HLA-B*27:05-positive versus HLA-B*27-negative subjects at R264X [Gag] and K903X [Pol] were 15 and 10, respectively). In contrast, strong immune selection pressure on the Nef VW9 and Pol KY9 epitopes was observed in HLA-B*27:02-positive subjects, and although escape mutation at R264X was observed in a minority of HLA-B*27:02-positive subjects (3 of 22 subjects studied), the selection of escape variants in Gag KK10 was clearly less frequent than in Nef VW9 or Pol KY9 (odds ratios for escape in HLA-B*27:02-positive versus HLA-B*27-negative subjects at R264X [Gag], R902X [Pol], and L137X [Nef] were 4, 15, and 15, respectively). Consistent with previous studies comparing the footprints of closely related HLA types on the same epitope (20, 21) we observed in Pol KY9 different footprints for HLA-B*27:02 (dominant footprint, R902X) and HLA-B*27:05 (dominant footprint, K903X).

**TABLE 2:**
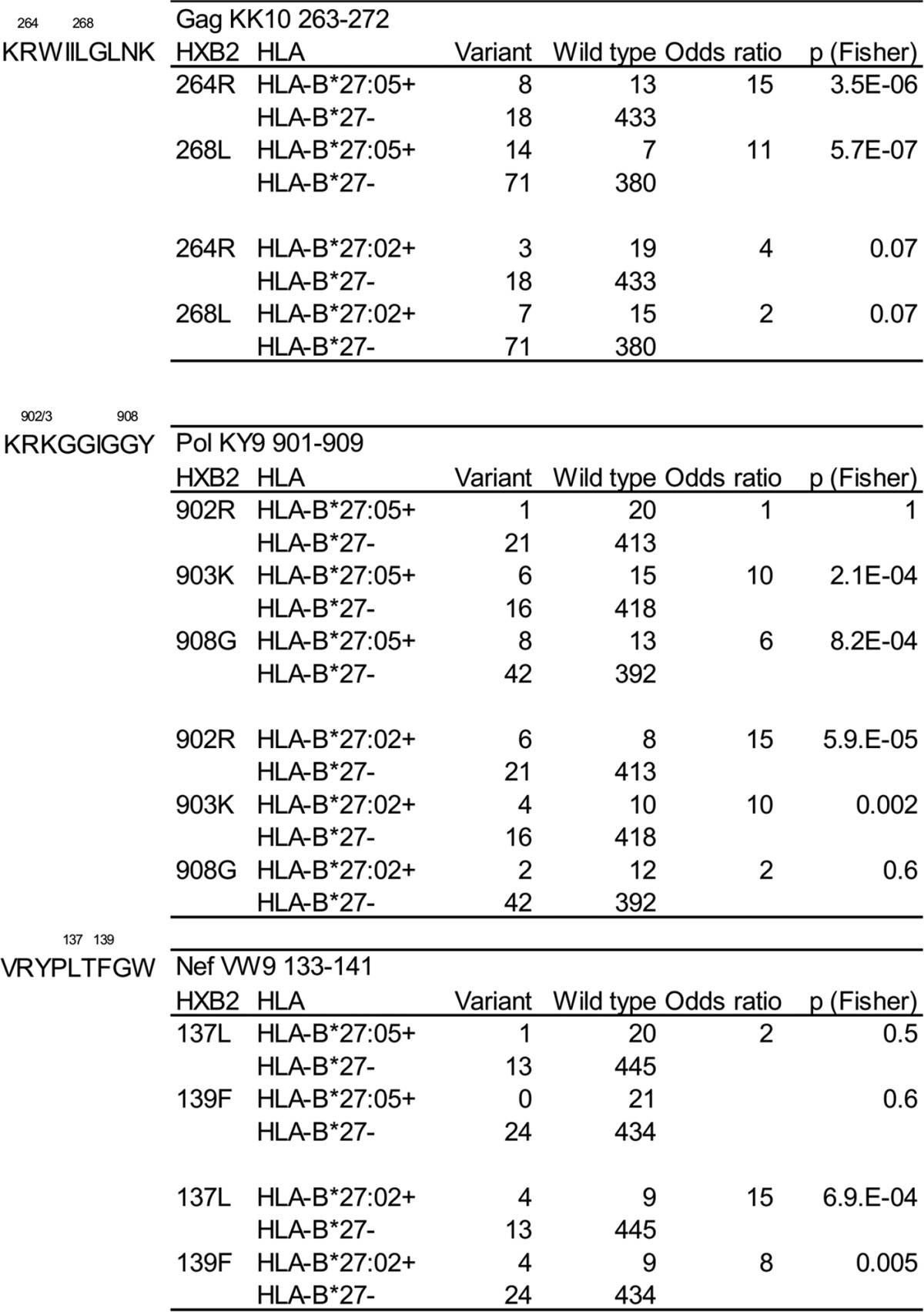
Analysis of selection pressure imposed by HLA-B*27:05- and HLA-B*27:02 positivity on immunodominant viral epitopes^*a*^

a Selection pressure on Gag KK10 (residues 263 to 272), Pol KY9 (residues 901 to 909), and Nef VW9 (residues 133 to 141) epitopes was examined in HLA-B*27:05-positive (*n* = 21), HLA-B*27:02-positive (*n* = 22), and HLA-B*27-negative (*n* = 458) subjects. *P* values were obtained from Fisher's exact test.

## DISCUSSION

These studies focus on HLA-B*27 and the mechanisms by which this molecule is associated with protection in HIV infection. We show that HLA-B*27:02 expression is associated with slower progression to HIV disease and also with lower viral loads than in the absence of expression of HLA-B*27. HLA-B*27:02 appears to be at least as protective as, and may be slightly more protective than, HLA-B*27:05. The immunodominant HLA-B*27:02-restricted HIV-specific CD8^+^ T-cell response is directed toward an epitope in Nef (VRYPLTFGW [residues 133 to 141]) that is not HLA-B*27:05 restricted. The two principal HLA-B*27:05-restricted epitopes, Gag KK10 and Pol KY9, are both also HLA-B*27:02 restricted, but Gag KK10 is the subdominant HLA-B*27:02-restricted response. These distinct HIV-specific CD8^+^ T-cell hierarchies are confirmed in the studies showing the selection of escape mutants within these epitopes. The strongest selection pressure in the HLA-B*27:02-positive subjects is for escape within Nef VW9 and Pol KY9, with weak pressure evident for escape within Gag KK10. These data suggest that while the immunodominant Gag KK10 response may play an important part in HLA-B*27:05-mediated immune control of HIV, access to the Nef VW9 epitope and alteration of the CD8^+^ T-cell immunodominance hierarchy in subjects expressing HLA-B*27:02 do not reduce HLA-B*27-associated protection against HIV disease progression.

As described above, the prevailing hypothesis is that HLA-B*27 is protective against HIV disease progression because the immunodominant response, Gag KK10, is highly efficacious, killing virus-infected target cells very soon after viral entry (8), and escape mutants are typically selected late in the course of infection (5) because of the crippling impact of the R264K or R264G mutation in the absence of a simultaneous compensatory mutation at S173T or E260D, respectively (22, 23). This hypothesis has been extended to explain HLA-B*57-mediated control also, a broad Gag-specific response (24) here being even more protective than that generated in HLA-B*27-positive subjects and multiple mutations required in several Gag epitopes, each likely to occur at a detriment to viral fitness (25, 26).

Although there are exceptions—HLA-B*14:02 and HLA-B*51:01 being two of these (27, 28)—most of the HLA-B alleles that have shown a well-documented association with favorable control of viremia, including HLA-B*27:05, HLA-B*57, HLA-B*58:01, HLA-B*13:02, HLA-B*52:01, and HLA-B*81:01 (29–32), have an immunodominant anti-HIV CD8^+^ T-cell response within p24 Gag. In general, HLA molecules associated with more rapid progression to AIDS, such as HLA-B*18:01, HLA-B*35:01, and HLA-B*58:02, show dominant responses directed at non-Gag epitopes such as Nef or Env (16, 24, 33–35). Here we show that the HLA-B*27:02 is at least as protective as HLA-B*27:05, and the immunodominant HIV-specific epitope is located in Nef. Although Nef targeting may not generally be associated with improved control of HIV (24, 36, 37), a study of simian immunodeficiency virus (SIV) infection in Mamu-B*08 rhesus macaques, an animal model for HLA-B*27-mediated elite control (38), showed that the frequency of the CD8^+^ T-cell response against a Nef epitope correlated significantly with reduced acute-phase viremia (39). This is one of the first models to demonstrate that a vaccine-induced Nef-specific CD8^+^ T-cell response can control replication of an AIDS virus in an animal model of MHC class I-associated control. Indeed, elite control of SIV in macaques expressing one of the protective MHC alleles, Mamu-B*08 and Mamu-B*17, have little or nothing in the way of Gag responses. Protective responses appear to lie exclusively in Nef or Vif. Indeed, it is striking that the immunodominant Mamu-B*17 epitope in Nef, IRYPKTFGW (40), corresponds exactly with the immunodominant HLA-B*27:02-restricted epitope described here, VRYPLTFGW. Mamu-B*17 in fact bears strong similarity with HLA-B*27:02 in binding peptides that carry Arg at P2 and Trp at PC (41). It seems remarkable and not coincidental that two MHC class I molecules that have evolved independently but, by convergent evolution (42), possess the ability to bind similar peptides can mediate, independently, control of SIV and HIV infection, respectively.

Thus, although immunodominant p24 Gag-specific immune responses are generally associated with effective immune control of HIV and Nef-specific CD8^+^ T-cell responses are not, this does not exclude the fact that certain non-Gag-specific CD8^+^ T-cell responses may also contribute to successful suppression of viral replication. Furthermore, as evidenced by the finding of escape mutations within Gag KK10, albeit in a minority if HLA-B*27:02-positive subjects, the Gag KK10-specific response may continue to contribute to control of HIV in HLA-B*27:02-positive subjects, even if it is not the dominant response. Numerous previous examples of subdominant responses being more efficacious in control of virus infections, including HIV, have been presented (43).

The reasons for the differences observed between HLA-B*27:05 and HLA-B*27:02 in the CD8^+^ T-cell immunodominance hierarchy were explored using structural modeling. These analyses demonstrated a clear structural difference within the F pockets of the two alleles, mainly attributed to the L81A polymorphism. The narrower F pocket in HLA-B*27:05 (governed by Leu-81), although ideally suited for binding to peptides with Lys at the C terminus, would likely be unable to accommodate the larger Trp side chain in the Nef VW9 peptide. On the other hand, the wider F pocket in HLA-B*27:02 (governed by Ala-81) would likely form a less stable interaction with Lys-10 but is ideally suited for interactions with peptides with Trp at the C terminus. The resulting low binding avidity of HLA-B*27:02 for Gag KK10 and high binding avidity for Nef VW9, and the converse for HLA-B*27:05, largely explain the altered immunodominance patterns and consequent escape hierarchies (19, 44) observed.

Comparisons between the CD8^+^ T-cell responses restricted by HLA-B*27:02 and HLA-B*27:05 have not been made previously in HIV infection, but a similar study has been undertaken with regard to hepatitis C virus (HCV) infection (45). As in HIV, HLA-B*27 is associated with improved HCV disease outcome (46, 47), and also as in HIV, HLA-B*27:02 appears to present more epitopes than HLA-B*27:05. Of three HLA-B*27-restricted HCV NS5B-specific epitopes, two are presented by both HLA-B*27:02 and HLA-B*27:05 (NS5B amino acid sequences ARMILMTHF and GRAAICGKY) and a third by HLA-B*27:02 only (amino acid sequence ARHTPVNSW). It is striking that the HLA-B*27:02-restricted epitope ARHTPVNSW carries Trp at PC, just as the Nef VW9 HLA-B*27:02-restricted epitope described here, which from the structural considerations described above would not be expected to be accommodated within the smaller F pocket of HLA-B*27:05. Also, it is notable that these HLA-B*27:02-specific epitopes, ARHTPVNSW and Nef VW9, in HCV and HIV, respectively, are both clearly the immunodominant responses among HLA-B*27:02-positive individuals. This is consistent with findings from comparisons of HLA-B*44:02 and HLA-B*44:03 (48), HLA molecules differing by only a single amino acid residue, demonstrating that the greater capacity within the HLA-B*44:03 peptide-binding groove allows a larger repertoire of peptides to bind. Also, structural studies of peptide binding to HLA-B*57:03 (18), which, like HLA-B*27:02, has Ala at position 81, show that large residues, such as Trp, binding in the appropriately sized F pocket make greatly increased numbers of interatomic van der Waals contacts that contribute to the stability of the peptide-MHC complex and therefore to immunodominance of the response.

The limitations of the current study include the fact that HIV-infected HLA-B*27:02-positive subjects were very hard to find and therefore only a relatively small number were studied. In addition, other than defining the specificity of the HIV-specific CD8^+^ T-cell responses and the seeking selection pressure on the virus through the three main specificities of interest, sample availability limited further analyses to investigate the ability of the HLA-B*27:02 Nef response to inhibit viral replication. In addition, the study has focused solely on the HLA-B*27-restricted CD8^+^ T-cell response, although it is known that HLA-mediated effects on HIV disease outcome may arise via other mechanisms (49–51). In particular, the finding that Bw4-80I-expressing alleles in combination with high-expression KIR3DL1 alleles are associated with more effective control of HIV (49) provides an additional potential explanation for the improved action of HLA-B*27:02 (a Bw4-80I allele) in comparison with HLA-B*27:05 (a HLA-Bw4-80T allele) in control of HIV.

In conclusion, despite the subdominance of Gag KK10 in HLA-B*27:02-positive subjects, HLA-associated protection against HIV disease progression is at least as strong as that mediated by HLA-B*27:05. The immunodominant Nef VW9-specific response may contribute to this additional immune control, in combination with contributions made via the Gag KK10 and Pol KY9 specificities that are shared with HLA-B*27:05. In addition, there may be additional mechanisms, such as the HLA-B*27:02 interaction with KIR3DL1, operating to supplement further the antiviral immune effects of HLA-B*27:02 against HIV.

## MATERIALS AND METHODS

### Study cohorts.

We studied treatment-naive subjects with chronic HIV-1 infection from (i) Warsaw, Poland, (ii) Athens, Greece, (iii) Mexico City, Mexico, (iv) Bonn, Germany, (v) Boston, MA, (vi) Barcelona, Spain, and (vii) Thames Valley, UK. Several cohorts were used for the study because of the paucity of HIV-infected subjects expressing HLA-B*27:02. Subjects were included in the study if they were HIV infected and HLA-B*27:02 positive and samples were available for either ELISpot assays or viral sequencing. The absolute CD4 count of these study subjects was 460 cells/μl (interquartile range [IQR], 287 to 647), and the median viral load was 11,399 copies/ml of plasma (IQR, 437 to 29,592). Study subjects from all cohorts gave written informed consent for their participation. The study was approved by the institutional review boards of the University of Oxford, University of Warsaw, Medical School, National and Kapodistrian University of Athens, University of Bonn, National Institute of Respiratory Diseases in Mexico City, Ragon Institute, and University Hospital Germans Trias i Pujol in Badalona (Barcelona, Spain).

For survival analyses, we included 783 individuals from five studies—AIDS Linked to the Intravenous Experience (ALIVE; *n* = 12) (52), the Multicenter AIDS Cohort Study (MACS; *n* = 417) (53), the Multicenter Haemophilia Cohort Study (MHCS; *n* = 243) (54), the San Francisco City Clinic Cohort (SFCCC; *n* = 74) (55), and the DC Gay Cohort Study (DCGCS; *n* = 37) (56)—with prospective follow-up and known dates of seroconversion. For HIV mean viral load analyses, we included 3,280 individuals enrolled in one of five prospective studies: the Multicenter AIDS Cohort Study (*n* = 1,583), the Military HIV Research Program (MHRP; *n* = 191), the Ragon Institute of MGH, MIT and Harvard HIV Controller study (*n* = 975), the Study of the Consequences of the Protease Inhibitor Era (SCOPE; *n* = 386), and the Swiss HIV Cohort study (*n* = 145). There was an overlap of 414 subjects between the two analyses.

Measures of disease outcome in the survival analyses of HIV-infected subjects were an absolute CD4 count of <200 cells/mm^3^, meeting the 1987 CDC definition of AIDS (AIDS defining illness), meeting the 1993 CDC definition of AIDS (AIDS defining illness) or decline to absolute CD4 count of <200 cells/mm^3^, and death.

The frequency of amino acid polymorphisms among HLA-B*27-negative, B-clade-infected individuals within the Gag, Pol, and Nef HLA-B*27 epitopes shown in Table 2 was determined from analysis of 555 AIDS Clinical Trials Group and 245 Western Australia HIV Cohort Study subjects (57).

### HLA typing.

*HLA* genotyping was performed by either PCR–sequence-specific oligonucleotide probing (PCR-SSOP), PCR–sequence-based typing (PCR-SBT) using the Sanger sequencing technology recommended by the 13th International Histocompatibility Workshop (http://www.ihwg.org), or next-generation sequencing using the Roche 454 platform (58).

### Amplification and sequencing of HIV genes by PCR.

Gag, Pol, and Nef sequences were generated from either viral RNA or genomic DNA. DNA was extracted from whole blood, and viral RNA was extracted from plasma using an RNA extraction minikit (Qiagen UK) in accordance with the manufacturer's instructions. Reverse transcription of RNA to cDNA was undertaken using a Superscript III one-step reverse transcriptase kit (Invitrogen) as a one-step reaction combined with outer PCR according to the manufacturer's instructions and amplified by nested PCR to obtain population sequences. Sequencing was undertaken using the BigDye Ready Reaction Terminator Mix (V3) (Applied Biosystems UK) analyzed using Sequencher v4.8 (Gene Codes Corporation) and manually aligned using Se_Al software.

### IFN-γ ELISpot assays.

We tested *ex vivo* peripheral blood mononuclear cells (PBMCs) against a panel of 410 overlapping peptides (OLPs) spanning the entire HIV-1 proteome to screen for IFN-γ ELISpot responses (14). We additionally tested putative optimal epitopes by ELISpot assay using *ex vivo* PBMCs from HLA-B*27:02- and HLA-B*27:05-positive subjects.

### Cell staining and flow cytometry.

Cell staining from cryopreserved PBMCs was undertaken using anti-CD3-Pacific orange (Invitrogen), anti-CD8-Alexa Fluor 700 (BD Biosciences), and HLA-B*27 tetramers conjugated to phycoerythrin (PE). Dead cells were gated out using a LIVE/DEAD viability kit (Invitrogen).

### Peptide–MHC-I binding assay.

Peptide affinity to HLA class I molecules was determined using a luminescent oxygen channeling immunoassay (LOCI) (59). Briefly, peptides were dissolved in phosphate-buffered saline (PBS)–0.1% Lutrol F68 by sonication for 10 min. Peptides were titrated in 384-well microplates using a Microlab STAR liquid handling robot (Hamilton Robotics). Recombinant, denatured HLA-C heavy chain (HC) was diluted into PBS–0.1% Lutrol F68/100 mM Tris/maleate (pH 6.6) containing prefolded, recombinant beta-2 microglobulin (β_2_m) on ice. The HC-β_2_m mix was added 1:1 to the peptide titrations and incubated for 48 h at 18°C to allow peptide–MHC-I complex folding. After complex folding, samples were transferred to 384-well Optiplates and streptavidin-coated donor beads (PerkinElmer; 6760002) and W6/32-conjugated acceptor beads (PerkinElmer; 6762001; in-house conjugated with W6/32) diluted in PBS–0.1% Lutrol F68 were added to a final concentration of 5 μg/ml each. The Optiplates were incubated overnight and luminescence was measured in an EnVision 2103 multilabel reader.

### Structural analysis.

The HLA-B*27:05 KK10 structure (PDB code 4G9D) (17) was used to model analyze C-terminal peptide residue interaction with the F pocket, in which Lys-10 was mutated to Trp-10. This structure was also used to model the F pocket of HLA-B*27:02 (D77N, T81I, and L81A) with both Lys-10 and Trp-10. Sequences were adjusted with COOT (60), and graphical representations were prepared with PYMOL (PyMOL molecular graphics system, version 1.8; Schrodinger, LLC).
